# Genotypes and Toxin Gene Profiles of *Staphylococcus aureus* Clinical Isolates from China

**DOI:** 10.1371/journal.pone.0028276

**Published:** 2011-12-15

**Authors:** Yanping Xie, Yiping He, Andrew Gehring, Yu Hu, Qiongqiong Li, Shu-I Tu, Xianming Shi

**Affiliations:** 1 School of Agriculture and Biology, Bor Luh Food Safety Center, Shanghai Jiao Tong University, Shanghai, China; 2 Molecular Characterization of Foodborne Pathogens Research Unit, U. S. Department of Agriculture, Agricultural Research Service, Eastern Regional Research Center, Wyndmoor, Pennsylvania, United States of America; Baylor College of Medicine, United States of America

## Abstract

A total of 108 *S. aureus* isolates from 16 major hospitals located in 14 different provinces in China were characterized for the profiles of 18 staphylococcal enterotoxin (SE) genes, 3 exfoliatin genes (*eta*, *etb* and *etd*), and the toxic shock syndrome toxin gene (*tsst*) by PCR. The genomic diversity of each isolate was also evaluated by pulsed-field gel electrophoresis (PFGE), multilocus sequence typing (MLST), and accessory gene regulator (*agr*) typing. Of these strains, 90.7% (98/108) harbored toxin genes, in which *tsst* was the most prevalent toxin gene (48.1%), followed by *sea* (44.4%), *sek* (42.6%) and *seq* (40.7%). The *see* and *etb* genes were not found in any of the isolates tested. Because of high-frequency transfer of toxin gene-containing mobile genetic elements between *S. aureus* strains, a total of 47 different toxin gene combinations were detected, including a complete *egc* cluster in 19 isolates, co-occurrence of *sea*, *sek* and *seq* in 38 strains, and *sec* and *sel* together in 11 strains. Genetic typing by PFGE grouped all the strains into 25 clusters based on 80% similarity. MLST revealed 25 sequence types (ST) which were assigned into 16 clonal complexes (CCs) including 2 new singletons. Among these, 11 new and 6 known STs were first reported in the *S. aureus* strains from China. Overall, the genotyping results showed high genetic diversity of the strains regardless of their geographical distributions, and no strong correlation between genetic background and toxin genotypes of the strains. For genotyping *S. aureus*, PFGE appears to be more discriminatory than MLST. However, toxin gene typing combined with PFGE or MLST could increase the discriminatory power of genotyping *S. aureus* strains.

## Introduction

*S. aureus* is one of the most common clinical and foodborne pathogens. It can cause a wide variety of infections ranging from skin and tissue infections, toxin-mediated diseases, pneumonia, and bacteremia. The infections of *S. aureus* can be acquired through both hospital and community settings including food poisoning. It has been reported that *S. aureus* colonizes the skin and mucosal surface of 20 to 40% of humans, implying a high risk of developing the infections of this organism [1]. A recent study found that 47% of meat and poultry in US grocery stores were contaminated with *S. aureus*, and 52% of those bacteria were resistant to antibiotics [2]. The high prevalence and rapid spread of drug-resistant *S. aureus* strains in food supplies have increased the risk of *S. aureus* infections and posed a great threat to food safety and public health.

*S. aureus* can produce a wide variety of virulence factors. Depending on the strain, some can produce exotoxins such as toxic shock syndrome toxin (TSST), a causative agent of toxic shock syndrome, and exfoliative toxins (ETs), responsible for staphylococcal scalded skin syndrome. Some strains express heat-stable enterotoxins (SEs), a major cause of staphylococcal food poisoning [3]. To date, there are 21 identified SE and staphylococcal-like enterotoxin (SE*l*) genes, including *sea* to *see*, *seg* to *sev*. Among them, SEA is the most common enterotoxin found in food and is frequently associated with staphylococcal food-poisoning outbreaks worldwide [4]. All of the SEs share sequence and structural similarities and are typically encoded by the genes located on mobile genetic elements (MGEs), such as plasmids, prophages, transposons, or pathogenicity islands. These MGEs are effective vehicles for spreading virulence and drug resistance genes between *S. aureus* strains through horizontal gene transfer, which often changes the ability of the pathogen to cause disease and has significant impact on the evolution of the organism [5]. It is known that the sequences of certain SE genes are grouped together on MGEs. For example, the enterotoxin gene cluster (*egc*), comprising *seg-sei-sem-sen-seo* and sometimes *seu*, is located on the genomic island vSAβ [6]. The genes of *sea-sek-seq* are typically present together in phage ϕ3 [5]. A family of pathogenicity islands carries *seb-sek-seq* on SaPI in *S. aureus* COL [6], *tsst-sec-sel* on SaPI2 (SaPln1/SaPlm1) in strain N315/Mu50 [6], [7], or *sec-sel* on SaPI3 in strain MW2 [6], [8]. In addition, *sed-sej-ser* are encoded on plasmids, and *seh* is linked to the staphylococcal cassette chromosome *mec* (SCC*mec*) elements, a determinant of methicillin resistance [5], [9].

As major virulence factors in *S. aureus*, TSST, ETs, and SEs have been implicated in host colonization, invasion of damaged skin and mucus, gastrointestinal infection, and evasion of host defense mechanisms. Therefore, it is important to determine the toxin gene profiles of *S. aureus* strains from different clinics to understand the genetic and pathogenic relatedness, as well as the epidemiology of *S. aureus*. While Wu *et al*. [10] have recently reported SE gene profiles of community-acquired methicillin-resistant *S. aureus* isolates (CA-MRSA) from Chinese children, few reports have described enterotoxin and exotoxin gene profiles of clinical *S. aureus* strains from different geographical areas in China, especially in methicillin-sensitive *S. aureus* (MSSA). Expression of most virulence factors in *S. aureus* is controlled by the accessory gene regulator (*agr*) locus. Based on the amino acid sequence polymorphisms of the *agr*-encoding autoinducing peptide and its corresponding receptor, *S. aureus* strains can be divided into 4 major *agr* groups (I – IV) [11]. It would be interesting to determine the prevalence of *agr* groups in *S. aureus* isolates from various hospitals and to investigate a possible relationship between *agr* groups and the occurrence of toxin genes.

For genetic typing of bacterial strains, pulse-field gel electrophoresis (PFGE) is known to be a highly discriminatory technique and is frequently used for characterizing genetic diversity and outbreak investigations of microbial pathogens. PFGE is a whole genome typing method based on DNA fragment patterns generated by restriction digestion, so its stability may be insufficient for reliable studies of the evolution and phylogenetic relationships of bacterial strains [12]. Despite recent improvements on standardized protocols and interpretation criteria of PFGE data, comparison of the results from different laboratories remains difficult. Multilocus sequence typing (MLST), which involves sequencing 7 housekeeping genes in each *S. aureus* genome and then comparing them with the established sequence information in MLST database (http://www.mlst.net), could be more accurate and reproducible. However, housekeeping genes are relatively stable by nature, and changes in these genes accumulate slowly over time, making MLST less discriminatory than PFGE [13]. Therefore, it would be advantageous to use both techniques for accurate and discriminatory genotyping of bacterial populations.

In this study, we examined 108 clinical *S. aureus* isolates from different regions of China for the patterns of 22 toxin genes by PCR. Genotypic information of the strains was determined by PFGE, MLST, and *agr* typing. The relationships between the genotypes and toxin gene profiles, genetic background and geographical distributions of the strains were analyzed. Finally, toxin gene typing was compared to PFGE and MLST methods for discriminatory ability in genotyping *S. aureus* strains.

## Results

### Toxin gene content in *S. aureus* isolates

**Figure 1:**
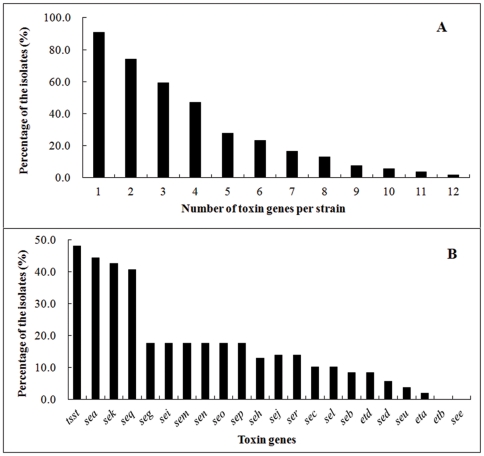
Prevalence of 22 toxin genes in 108 *S. aureus* clinical isolates. (A) The percentage of isolates containing different numbers of (1 to 12) toxin genes per strain. (B) The occurrence of each toxin gene in the isolates.

By PCR amplification of sequence-specific regions in 22 toxin genes, the occurrence of the SE, ET and TSST genes in 108 *S. aureus* isolates was determined. It was found that 98 strains (90.7%) contained at least one toxin gene and 80 strains (74.1%) carried two or more toxin genes (Fig. 1A). Although the average number of toxin genes per isolate was 4, up to 12 distinct toxin genes were detected in two given strains. The prevalence of each SE gene, *eta*, *etb*, *etd*, and *tsst* in the isolates is shown in Fig. 1B. The most abundant toxin gene was *tsst* presented in 52 (48.1%) isolates, followed by *sea* (44.4%), *sek* (42.6%), and *seq* (40.7%). The *see* and *etb* genes were not detected in any of the isolates. Consistently, *see* was reported to be the least frequently identified SE gene in *S. aureus* [14].

In 98 toxin gene-positive strains, there were 47 different toxin genotypes, of which 6 genotypes contained a single toxin gene and the rest were comprised of multiple toxin genes (Table 1). Notably, the combinations of SE genes were conserved in these strains. The *sea-sek*-*seq* combination including *sea-sek*-*seq* co-existed with additional toxin genes was the most common, harbored by 38 isolates. The *seb-sek-seq* combination was in 5 isolates, and a complete set of *egc* clusters (*seg, sei, sem, sen* and *seo*) was found in 19 isolates, of which 4 contained an additional SE gene (*seu*). The *sej*-*ser* and *sed*-*sej*-*ser* genes were present in 15 and 5 isolates, respectively, and *sec*-*sel* was found in 11 isolates. Previously, *sea-sek-seq* and *seb-sek-seq*, carried by staphylococcal phage ϕ3 and pathogenicity island SaPI1, respectively, were reported to coexist in *S. aureus* [10]. In agreement, this study detected *sea-seb-sek-seq* together in two of the isolates. Moreover, different combinations of *sea-sek-seq, sec-sel, sed-sej-ser*, and *egc* clusters were found in these strains. The high prevalence and conserved combinations of SE genes in these clinical strains suggest a high-frequency transfer of MGEs encoding various SE genes, which could significantly contribute to pathogenicity of these strains.

**Table 1:** Toxin genotypes of *S. aureus* isolates in different clonal complexes.

	Clonal Complex																
	1	5	6	7	8	15	20	25	30	59	88	121	188	239	1922	1926	Total
Toxin Genotype	Number of Isolates																
Negative for toxin genes	1	-	-	-	3	3	-	-	-	-	1	-	2	-	-	-	10
*sea*	-	-	2	-	-	-	-	-	-	-	-	-	-	-	-	-	2
*seb*	-	-	-	-	-	-	-	-	-	-	-	-	1	-	-	-	1
*sep*	-	-	-	5	1	-	-	-	-	-	1	-	-	-	-	-	7
*seh*	1	-	-	-	-	-	-	-	-	1	-	-	-	-	-	-	2
*tsst*	-	-	-	-	-	1	-	-	-	1	-	-	2	-	-	-	4
*etd*	-	-	-	-	-	-	-	1	-	-	-	-	1	-	-	-	2
*sea, sek*	-	-	-	-	-	-	-	-	-	-	-	-	-	1	-	-	1
*sea, sep*	-	-	-	-	-	1	-	-	-	-	-	-	-	-	-	-	1
*sej, ser*	-	-	-	-	-	1	-	-	-	-	-	-	-	-	-	-	1
*sek, seq*	-	-	-	-	-	-	-	-	-	-	-	-	-	1	-	-	1
*sep, etd*	-	-	-	1	-	-	-	-	-	-	-	-	-	-	-	-	1
*tsst, seb*	-	-	-	-	-	-	-	-	-	-	-	1	1	-	-	-	2
*tsst, sep*	-	-	-	2	-	-	-	-	-	-	4	-	-	-	-	1	7
*tsst, seh*	2	-	-	-	-	-	-	-	-	-	-	-	-	-	-	-	2
*sea, sek, seq*	-	-	-	-	-	-	-	-	-	-	-	-	-	11	-	-	11
*seb, sek, seq*	-	-	-	-	-	-	-	-	-	1	-	-	-	-	-	-	1
*tsst, sea, eta*	-	-	1	-	-	-	-	-	-	-	-	-	-	-	-	-	1
*sea, sek, seq, etd*	-	-	-	-	-	-	-	-	-	-	-	-	-	2	-	-	2
*sea, sek, seq, seh*	2	-	-	-	-	-	-	-	-	-	-	-	-	1	-	-	3
*tsst, sec, sel, etd*	-	-	-	-	-	-	-	-	-	-	-	-	-	-	1	-	1
*tsst, sea, sek, seq*	-	-	-	-	-	-	-	-	-	-	-	-	-	13	-	-	13
*tsst, sea, sek, seh*	-	-	-	-	-	-	-	-	-	-	-	-	-	1	-	-	1
*tsst, sek, seq, seh*	1	-	-	-	-	-	-	-	-	-	-	-	-	-	-	-	1
*sea, seb, sek, seq, seh*	1	-	-	-	-	-	-	-	-	-	-	-	-	-	-	-	1
*seb, sek, seq, sep, etd*	-	-	-	-	-	-	-	-	-	1	-	-	-	-	-	-	1
*tsst, sea, sed, sek, seq*	-	-	-	-	-	-	-	-	-	-	-	-	-	1	-	-	1
*tsst, sea, sek, seq, seh*	1	-	-	-	-	-	-	-	-	-	-	-	-	-	-	-	1
*tsst, seb, sek, seq, seh*	-	-	-	-	-	-	-	-	-	1	-	-	-	-	-	-	1
*sea, seg, sei, sem, sen, seo*	-	2	-	-	-	-	-	-	-	-	-	-	-	-	-	-	2
*tsst, sea, sek, seq, sej, ser*	-	-	-	-	-	-	-	-	-	-	-	-	-	4	-	-	4
*seg, sei, sem, sen, seo, seu*	-	-	-	-	-	-	-	-	-	-	-	1	-	-	-	-	1
*sej, ser, seg, sei, sem, sen, seo*	-	-	-	-	-	-	1	-	-	-	-	-	-	-	-	-	1
*tsst, sea, seg, sei, sem, sen, seo*	-	-	-	-	-	-	-	-	1	-	-	-	-	-	-	-	1
*tsst, sea, sek, seq, seh, sec, sel*	2	-	-	-	-	-	-	-	-	-	-	-	-	-	-	-	2
*sed, sej, ser, seg, sei, sem, sen, seo*	-	1	-	-	-	-	-	-	-	-	-	-	-	-	-	-	1
*sej, ser, seg, sei, sem, sen, seo, sep*	-	1	-	-	-	-	-	-	-	-	-	-	-	-	-	-	1
*tsst, seg, sei, sem, sen, seo, seu, eta*	-	-	-	-	-	-	-	-	-	-	-	1	-	-	-	-	1
*tsst, sec, sel, seg, sei, sem, sen, seo*	-	2	-	-	-	-	-	-	-	-	-	-	-	-	-	-	2
*tsst, sej, ser, seg, sei, sem, sen, seo*	-	-	-	-	-	1	-	-	-	-	-	-	-	-	-	-	1
*sea, sed, sej, ser, seg, sei, sem, sen, seo*	-	1	-	-	-	-	-	-	-	-	-	-	-	-	-	-	1
*tsst, sec, sel, seg, sei, sem, sen, seo, sep*	-	1	-	-	-	-	-	-	-	-	-	-	-	-	-	-	1
*sec, sel, sed, sej, ser, seg, sei, sem, sen, seo*	-	1	-	-	-	-	-	-	-	-	-	-	-	-	-	-	1
*tsst, seb, sej, ser, seg, sei, sem, sen, seo, etd*	-	-	-	-	-	-	-	1	-	-	-	-	-	-	-	-	1
*tsst, sec, sel, sed, sej, ser, seg, sei, sem, sen, seo*	-	1	-	-	-	-	-	-	-	-	-	-	-	-	-	-	1
*tsst, sec, sel, sej, ser, seg, sei, sem, sen, seo, seu*	-	-	-	-	-	-	-	-	1	-	-	-	-	-	-	-	1
*tsst, sec, sel, sed, sej, ser, seg, sei, sem, sen, seo, etd*	-	1	-	-	-	-	-	-	-	-	-	-	-	-	-	-	1
*tsst, seb, sek, seq, sec, sel, seg, sei, sem, sen, seo, seu*	-	-	-	-	-	-	-	-	1	-	-	-	-	-	-	-	1

Except for *seb* and *seu* found only in MSSA, the rest SE genes were present in both MRSA and MSSA. Interestingly, all of the MRSA strains were determined to be SE gene-positive, suggesting these MRSA strains could be more pathogenic than the MSSA strains.

### PFGE

**Figure 2:**
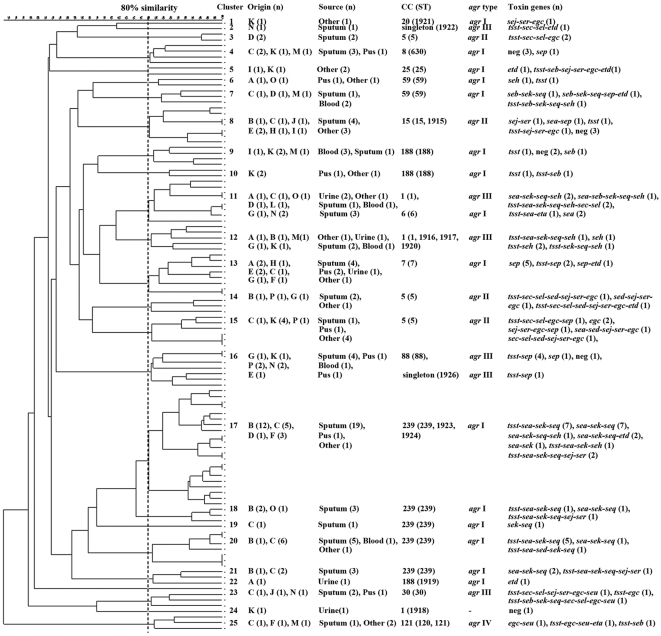
Dendrogram of PFGE clusters and genotypic relationships of *S. aureus* isolates. Based on PFGE patterns, 108 clinical isolates were grouped into 25 clusters. In each cluster, the hospital origins, clinical sample sources, MLST clonal complexes, *agr* types, and toxin gene contents of the strains are listed. *egc*: *seg-sei-sem-sen-seo* and neg: negative for toxin genes.

In 108 *S. aureus* isolates typed by PFGE, 25 clusters were classified by using 80% similarity as a cutoff. Each cluster was designated by a number from 1 to 25 (Fig. 2). In each cluster, the number of strains varied substantially, e.g., cluster 1, 2, 22 and 24 included only one strain, whereas cluster 17 contained 21 strains. The correlation between PFGE genotypes and strain origins was insignificant. Some isolates from the same source and location were typed into different PFGE clusters, e.g., 16 strains isolated from sputum from the First Hospital of Jinlin University were distributed to 9 different clusters. In contrast, some isolates from different origins and sources were grouped into the same PFGE clusters, e.g., 21 isolates from 5 hospitals and 3 different sources all belonged to cluster 17. Among the 25 PFGE clusters, 18 clusters included isolates from two or more different hospitals and 17 clusters had isolates from two or more sample sources. Furthermore, 39 MRSA isolates belonged to 6 different clusters and 69 MSSA strains were distributed to 19 different clusters, suggesting no association between methicillin resistance and PFGE type in these strains.

### MLST

As different sequences of each housekeeping gene were considered distinct alleles in MLST scheme, there were 7 new alleles identified in these isolates and assigned GenBank accession numbers of JN585753 (*aroE* allele 288), JN585754 (*glpF* allele 257), JN585755 (*gmk* allele 157), JN585756 (*tpi* allele 217), JN585757 (*tpi* allele 218), JN585758 (*yqiL* allele 218), and JN585759 (*yqiL* allele 219).

By MLST, 108 *S. aureus* isolates were grouped into 25 sequence types (STs) which were assigned to 16 clonal complexes (CCs) including two new singletons (Table 2). Among these, 11 STs (1915–1924, 1926) were submitted as new registrations to the MLST database (http://www.mlst.net) because of the sequence information from these new alleles. With the exception of ST120 and new STs, the rest of STs included more than one isolate. CC239 was a major MLST type which consisted of ST239 and two new STs (ST1923 and ST1924), and included 35 isolates. In agreement with the results in MLST database, all of the 35 isolates in CC239 were MRSA. The remaining 4 MRSA strains belonged to CC5. In contrast, 69 MSSA strains were distributed in 14 different CCs, including 9 new STs. In this study, not only were11 new STs identified, but also 6 known STs (ST6, 7, 15, 25,120 and 630) were first discovered in *S. aureus* isolates from China.

**Table 2:** *Staphylococcus aureus* clonal complexes and occurrence of toxin genes**.**

MLST Grouping		Number of Isolates Containing Toxin Genes																				
Clonal Complex	Seq Type	*tsst*	*sea*	*seb*	*sec*	*sed*	*seg*	*seh*	*sei*	*sej*	*sek*	*sel*	*sem*	*sen*	*seo*	*sep*	*seq*	*ser*	*seu*	*eta*	*etd*	ND*
CC1	ST1	4	6	1	2	-	-	7	-	-	7	2	-	-	-	-	7	-	-	-	-	-
	ST1916	1	-	-	-	-	-	1	-	-	-	-	-	-	-	-	-	-	-	-	-	-
	ST1917	-	-	-	-	-	-	1	-	-	-	-	-	-	-	-	-	-	-	-	-	-
	ST1918	-	-	-	-	-	-	-	-	-	-	-	-	-	-	-	-	-	-	-	-	1
	ST1920	1	-	-	-	-	-	1	-	-	-	-	-	-	-	-	-	-	-	-	-	-
CC5	ST5	5	3	-	6	5	11	-	11	6	-	6	11	11	11	2	-	6	-	-	1	-
CC6	ST6	1	3	-	-	-	-	-	-	-	-	-	-	-	-	-	-	-	-	1	-	-
CC7	ST7	2	-	-	-	-	-	-	-	-	-	-	-	-	-	8	-	-	-	-	1	-
CC8	ST630	-	-	-	-	-	-	-	-	-	-	-	-	-	-	1	-	-	-	-	-	3
CC15	ST15	2	1	-	-	-	1	-	1	1	-	-	1	1	1	1	-	1	-	-	-	3
	ST1915	-	-	-	-	-	-	-	-	1	-	-	-	-	-	-	-	1	-	-	-	-
CC20	ST1921	-	-	-	-	-	1	-	1	1	-	-	1	1	1	-	-	1	-	-	-	-
CC25	ST25	1	-	1	-	-	1	-	1	1	-	-	1	1	1	-	-	1	-	-	2	-
CC30	ST30	3	1	1	2	-	3	-	3	1	1	2	3	3	3	-	1	1	2	-	-	-
CC59	ST59	2	-	3	-	-	-	2	-	-	3	-	-	-	-	1	3	-	-	-	1	-
CC88	ST88	4	-	-	-	-	-	-	-	-	-	-	-	-	-	5	-	-	-	-	-	1
CC121	ST120	-	-	-	-	-	1	-	1	-	-	-	1	1	1	-	-	-	1	-	-	-
	ST121	2	-	1	-	-	1	-	1	-	-	-	1	1	1	-	-	-	1	1	-	-
CC188	ST188	3	-	2	-	-	-	-	-	-	-	-	-	-	-	-	-	-	-	-	-	2
	ST1919	-	-	-	-	-	-	-	-	-	-	-	-	-	-	-	-	-	-	-	1	-
CC239	ST239	19	32	-	-	1	-	1	-	4	33	-	-	-	-	-	31	4	-	-	-	-
	ST1923	-	1	-	-	-	-	-	-	-	1	-	-	-	-	-	1	-	-	-	1	-
	ST1924	-	1	-	-	-	-	1	-	-	1	-	-	-	-	-	1	-	-	-	1	-
Singletons	ST1922	1	-	-	1	-	-	-	-	-	-	1	-	-	-	-	-	-	-	-	1	-
	ST1926	1	-	-	-	-	-	-	-	-	-	-	-	-	-	1	-	-	-	-	-	-
**Total # of Isolates**		**52**	**48**	**9**	**11**	**6**	**19**	**14**	**19**	**15**	**46**	**11**	**19**	**19**	**19**	**19**	**44**	**15**	**4**	**2**	**9**	**10**

*ND: no detectable for toxin genes.

### *agr* typing

Four *agr* groups (I – IV) were detected in 108 *S. aureus* isolates by multiplex PCR based on the amplicon size difference. The results showed that *agr* group I was the most predominant, detected in 60.2% (65/108) of the strains. The groups of *agr* III, II, and IV were less common and found in 19.4%, 16.7%, and 2.8% of the isolates, respectively. One isolate repeatedly yielded negative result for any of the *agr* types tested.

### Association of toxin genes with genotypic background

When analyzing the correlation between toxin gene profiles and genotypes of PFGE, MLST, and *agr* typing of the strains, 47 toxin gene patterns were found in 108 *S. aureus* isolates classified into 25 PFGE clusters, 16 MLST CCs, and 4 *agr* groups (Fig. 2). Interestingly, diverse toxin gene patterns were found in the strains belonging to the same PFGE clusters, STs, and *agr* groups. For each cluster, the number of toxin genes could vary significantly from 1 to 12. For example, an MSSA strain belonging to CC30, PFGE cluster 23 and *agr* III carried an unusually large number (n = 12) of toxin genes. Likewise, 10 isolates in CC5, *agr* II and 3 different PFGE clusters were found to have 8 different SE gene patterns with high numbers of toxin genes (mean, 8.5; range, 6 to 12), all of which carried a complete *egc* cluster. However, the most common MLST type (C239, n = 35), present in *agr* I and 5 divergent PFGE clusters, had only 4 distinct SE genes patterns with lower numbers of toxins (mean, 3.8; range, 2 to 6).

Moreover, the most prevalent SE gene combination was *sea-sek-seq*, carried by 38 *S. aureus* isolates with 32 strains in CC239 and 6 strains in CC1. The combination of *seb-sek-seq* was found in 5 strains belonging to CC59 (3), CC1 (1) and CC30 (1), 6 of the 11 strains containing *sec*-*sel* belonged to CC5, and the remaining strains were in CC1 (2), CC30 (2) and singleton1922. All of the 5 isolates containing *sed-sej-ser* belonged to CC5. The *tsst* gene co-existed with different kinds of SE genes and was detected in various CCs (Table 1). Even though 28/52 *tsst* positive and 7/9 *etd* containing strains were found in *agr* I, the correlation between the *agr* types and occurrence of *tsst* or *etd* is insignificant because a majority (>60%) of the isolates belongs to *agr* group I.

Taken together, these data suggest that the toxin gene patterns do not strongly associate with strain genotypes determined by PFGE, MLST or *agr* typing, except that a majority (71.1%) of *sea-sek-seq* positive isolates were from two hospitals (B &C) in northern China and typed into CC239, *agr* I and PFGE clusters 17–21. Since more than one distinct toxin gene combination was found in each cluster, CC and *agr* group, toxin gene typing could be more discriminatory than PFGE, MLST or *agr* typing.

### Genetic diversity vs. geographical distribution

In 63 strains isolated from sputum from 14 different hospitals, 58 were toxin gene-positive strains which had 25 toxin genotypes and belonged to 13 MLST CCs and 18 PFGE clusters. The remaining 5 were toxin gene-negative strains belonging to 3 CCs and 4 PFGE clusters. Even in those strains isolated from sputum from the same hospital, there were several different genotypes, e.g., 16 isolates from sputum from Beijing Hospital had 6 toxin genotypes belonging to 4 CCs and 4 PFGE clusters. Similarly, the isolates from blood from 6 hospitals and pus from 7 hospitals both contain 7 toxin genotypes belonging to 5 and 8 MLST CCs, respectively. These results suggest a high genetic diversity among the *S. aureus* clinical isolates regardless of the geographical distributions or sources of the samples.

## Discussion

In this study, a collection of 108 clinical isolates from China were investigated for the presence of 18 SE and 3 exfoliatin genes, and the toxic shock syndrome toxin gene by PCR and were also genotyped by PFGE, MLST and *agr* typing. High genetic diversity of the strains was demonstrated by all of the typing methods. Although only 108 *S. aureus* isolates were analyzed, they were collected from patients in 16 major hospitals geographically distributed in 14 different provinces across China. We believe these results represent the prevalence of different *S. aureus* strains in China.

The occurrence of toxin genes in these isolates was found to be abundant and diverse as 90.7% of the isolates, including both MRSA and MSSA, carried toxin genes with a maximum number of 12 toxin genes per strain and a total of 47 unique toxin genotypes. Previous studies showed *seb* and *ser* were the most abundant toxin genes in the clinical *S. aureus* isolates from children in China and patients in New York State, respectively [10], [14]. We found *tsst* was the most common toxin gene existing in 52 (48.1%) isolates. This discrepancy is most likely due to the different origins of the strains. Furthermore, the most common combination of SE genes *sea-sek-seq*, harbored by 38 *S. aureus* isolates, of which 32 in CC239 were all MRSA, is in agreement with other reports [10]. Usually, *sea*-*sek*-*seq* is clustered in phage ϕSa3mu, whereas the *seb*-*sek*-*seq* cluster is in pathogenicity island SaPI1. In the present study, two strains in CC1 were found to contain *sea-seb-sek-seq*, indicating the possible co-existence of ϕSa3mu and SaPI1 in these isolates. In addition, *sea* or *seb* without *sek-seq*, and *sea-sek* without *seq* were detected in some strains. The findings of these uncharacterized toxin gene combinations imply that new variants on the mobile elements of ϕSa3mu or SaPI1 or the existence of additional MGEs carrying SE genes.

The *egc* cluster, present in the *S. aureus* genomic island vSAβ, has been reported to be strictly linked to the clonal background regardless of geographical distribution of the strains [6]. Previously, the *egc* cluster has been reported to be present in CC5, CC22, and CC45 isolates but not in CC8, CC12, CC15, and CC395 [10], [15]. This study found *egc* in 19 *S. aureus* isolates, with 11 of them in CC5 and the rest distributed to 5 MLST CCs including CC15 (Table 1). Based on these results, the *egc*-containing genomic island is not completely absent in CC15 and may transfer among different CCs. In addition, we found 2 *seb-egc* positive isolates, one in each of CC25 and CC30, which is different from the results of Varshney *et al*. [14] who reported that *seb*-positive *S. aureus* strains did not carry *egc*. The *tsst* gene, carried by pathogenicity island SaPI2, was found to co-exist with the *egc* cluster in 11 isolates, with 5 belonging to CC5, which is in agreement with the results of Takano *et al*. [16]. Also, *tsst* was present in 10 of 11 *sec-sel* positive isolates. Together, these findings support the frequent transfer and multiple occurrence of toxin gene-containing MGEs in *S. aureus* isolates.

The *seh* gene is reported to be restricted to the CC1 genomic background [5]. It was located in close proximity of the non-*mecA* containing SCC element harbored by a MSSA strain as well as the SCC*mec* type IV of *S. aureus* [17]. Previously in China and Taiwan, *seh*-positive isolates were found in CC1 and ST338 of CC59, but not in ST59 [10], [18]. Our results showed 10 *seh*-positive isolates in CC1, 2 in ST59 of CC59, and 2 in CC239 turned out to be MRSA. It suggests that all the *seh*-positive isolates, except for two in ST239, carried the non-*mecA* containing SCC element.

The association between specific *agr* groups and types of toxin production was reported contradictorily. Jarraud *et al.* [19] reported that *agr* IV strains were associated with exfoliative syndrome and most TSST producing strains belong to *agr* group III [20]. However, recent studies indicated no statistical association between *agr* groups and toxin types [21], [22]. Similarly, no strong correlation between *agr* groups and toxin types was obtained from this study. As the *agr* locus belongs to the core variable genome and was reported to be strongly linked with clonal lineages [6], [23], in agreement with that, our results showed that each CC exclusively belongs to one *agr* group.

It has been suggested that combined assessment of virulence gene profiles and genetic background could increase the discriminatory ability of genetic investigations of *S. aureus* strains from geographically diverse locations [14], [15]. Campbell *et al*. [24] found that *S. aureus* strains from different geographic regions had different profiles of virulence genes. Our results showed high diversity of toxin gene profiles and genotypes classified by PFGE and MLST. No significant correlation between the genotypes and geographic locations of the strains was shown, except that 71.1% *sea*-*sek*-*seq* positive strains came from two hospitals in northern China (Beijing Hospital and the First Hospital of Jinlin University) and were typed into PFGE clusters 17–21 and MLST CC239 (Fig. 2). Compared to the MLST database (http://www.mlst.net), 11 new STs and 6 known STs (6, 7, 15, 25,120 and 630) were first reported in *S. aureus* strains from China, which expands *S. aureus* genotype information in the database.

According to the typing results in Fig. 2, several isolates belonging to the same CCs were assigned to different PFGE clusters. For example, CC239 strains were present in 5 divergent PFGE clusters, the strains in each of CC1, CC5 or CC188 were distributed to 3 different PFGE clusters, and CC59 strains were assigned to two PFGE clusters. In contrast, the strains within the same PFGE clusters generally belonged to the same MLST CCs. These results suggest that PFGE may have better discriminatory power than MLST for typing *S. aureus* strains. However, both PFGE and MLST were less discriminatory than toxin genotyping since more than one toxin gene pattern was detected in each PFGE cluster or MLST CC for almost all of the SE-positive strains. In agreement, Varshney *et al.* [14] also pointed out that MLST, spa typing, and PFGE were all less discriminatory than SE content in *seb*-positive strains in the U.S. There were 28 distinct combinations of SE genes found in *S. aureus* strain USA300, the predominant cause of CA-MRSA infections in the U.S. [25]. We found 47 unique toxin gene combinations in 108 strains belonging to 25 PFGE clusters, 16 MLST CCs, and 4 *agr* types. Given that most strains contained a distinct toxin gene profile even if their MLST backgrounds or PFGE patterns were clonal, acquisition or loss of MGEs containing SE genes may occur frequently within individual lineages.

In conclusion, our results showed a high genetic diversity of the clinical *S. aureus* isolates from China. Toxin genotypes were highly diverse in these strains, which could be used as a discriminatory method in combination of PFGE or MLST for genotyping *S. aureus* strains. The combination of toxin genes was found to not restrict to genetic background (CC) of the strain, suggesting frequent transfer of toxin gene-containing MGEs within or among CCs. In this study, 11 new STs were submitted to the MLST database and 6 known STs were first reported in *S. aureus* strains from China, which significantly expands our current knowledge of genetic background of *S. aureus* strains in China. The new SE gene combinations identified herein suggest the existence of variants or new types of MGEs.

## Materials and Methods

### Ethics statement

We did not feel that ethics approval of human participants was necessary for this study since all of the *S. aureus* isolates were given as a gift from the Institute of Clinical Pharmacology at Peking University. This research did not involve in any clinical samples or human participants.

### Bacterial isolates and growth conditions

A total of 108 *S. aureus* isolates were originated from 16 hospitals located in 14 different provinces of China. Of these, 63 *S. aureus* strains were isolated from sputum, 10 from pus, 9 from blood, 6 from urine, and 20 from other clinical samples by the Institute of Clinical Pharmacology at Peking University (Table 3). All of the isolates were identified as *S. aureus* strains using the API Staph-Ident system (bioMerieux, Shanghai, China), and confirmed by16s RNA sequencing using previously described primers: F27 (5′-AGAGTTT-GATCCTGGCTCAG-3′) and R1492 (5′-TACGGTTACCTTGTTACGACTT-3′) [26]. Phenotypic detection of methicillin resistance of the strains was performed as described by Fan *et al.* showed that 39 strains were resistant to methicillin (MRSA) [27]. All of the *S. aureus* strains were routinely grown in tryptic soy broth (Becton Dickinson, Sparks, MD) overnight at 37°C.

**Table 3:** Information for *S. aureus* clinical isolates.

Strain origin			
Province	Hospital (Abbreviation)	Source (n^a^)	MRSA
Beijing	Peking University Hospital (A)	Sputum (3), Urine (2), Pus (1)	-**^b^**
	Beijing Hospital (B)	Sputum (16), Other (3)	17
Jilin	The First Hospital of Jilin University (C)	Sputum (16), Pus(1), Blood (1), Urine (1), Other (4)	15
Liaoning	The First Hospital of China Medical University (D)	Sputum (3), Blood (1), Other (1)	3
Tianjin	Tianjin Medical University General Hospital (E)	Sputum (2), Pus(2), Other (1)	-
Hebei	The Second Hospital of Hebei Medical University (F)	Sputum (3), Pus (1), Other (1)	3
Jiangsu	Jiangsu Province Hospital (G)	Sputum (4), Urine (1)	-
Shanghai	Zhongshan Hospital Fudan University (H)	Sputum (1), Other (1)	-
Zhejiang	The First Hospital of Zhejiang University (I)	Sputum (2), Other (1)	-
Guangdong	Shenzhen People's Hospital (J)	Sputum (2)	-
Hubei	Renmin Hospital of Wuhan University (K)	Sputum (3), Blood (2), Urine(2), Pus(2), Other (5)	-
Hunan	Xiangya Hospital Central-south University (L)	Blood (1)	-
Sichuan	West China Hospital Sichuan University (M)	Blood (3), Pus (1), Other (1)	-
	Chengdu Children's Hospital (N)	Sputum (3), Pus (2), Blood (1)	-
Chongqing	Southwest Hospital (O)	Sputum (2), Other (1)	1
Shanxi	Xijing Hospital (P)	Sputum (3), Other (1)	-

a: the number of isolates.

b: “-” refers to MSSA.

### PCR detection of staphylococcal toxin genes

Genomic DNA of *S. aureus* was purified using a modified cetyltrimethylammonium bromide method [28] and then was subjected to PCR amplification of 18 SE genes, 3 exfoliatin genes, and the *tsst* gene by using the primers listed in Table 4. All of the PCR were performed in a singleplex platform in a GeneAmp PCR system 9700 (Applied Biosystems, Foster City, CA). For each reaction, 1 µl of genomic DNA (approx. 100 ng) was added to a 24-µl PCR mixture containing 1U of Taq DNA polymerase (Fermentas Inc., Glen Burnie, MD), 1×buffer, 1.5 mM MgCl_2_, and 0.4 µM each primer. The amplified DNA fragments were separated in a 1.5% agarose gel stained with ethidium bromide. To ensure the primer specificity and PCR reliability, both positive and negative controls were included into each run of PCR. The toxin gene-positive strains used in the experiment were: *S. aureus* ATCC8095 positive for *sea*, *sed*, *sej*, *sek*, *seq*, and *ser*; *S. aureus* ATCC14458 for *seb*; *S. aureus* ATCC27664 for *see*; *S. aureus* ATCC27661 for *seg*, *sei*, *sel*, *sem*, *sen*, and *seo*. For *tsst*, *sec*, *seh*, *sel*, *seu*, *eta*, *etb*, and *etd*, *S. aureus* isolates confirmed by sequencing of these genes were used as positive controls. For a negative control, ddH_2_O was used instead of a DNA sample. To confirm the specificity of detecting toxin genes, PCR products from 10% of the toxin gene-positive samples were randomly selected for DNA sequencing, and the resulting nucleotide sequences were searched against the *S. aureus* sequence database in the GenBank using BLAST (http://www.ncbi.nlm.nil.gov).

**Table 4:** Primers used for PCR amplification of toxin genes in *S. aureus*.

Gene	Primer	Sequence (5′→3′)	Amplicon size (bp)	Reference
***sea***	Sea-F	ATTAACCGAAGGTTCTGTAGA	552	[28]
	Sea-R	TTGCGTAAAAAGTCTGAATT		
***seb***	Seb-F	TGTATGTATGGAGGTGTAAC	270	[32]
	Seb-R	ATAGTGACGAGTTAGGTA		
***sec***	Sec-F	ACCAGACCCTATGCCAGATG	371	[33]
	Sec-R	TCCCATTATCAAAGTGGTTTCC		
***sed***	Sed-F	CTAGTTTGGTAATATCTCCT	317	[34]
	Sed-R	TAATGCTATATCTTATAGGG		
***see***	See-F	TAGATAAAGTTAAAACAAGC	170	[34]
	See-R	TAACTTACCGTGGACCCTTC		
***seg***	Seg-F	CCACCTGTTGAAGGAAGAGG	432	[33]
	Seg-R	TGCAGAACCATCAAACTCGT		
***seh***	Seh-F	CACATCATATGCGAAAGCAGA	617	This study
	Seh-R	CCTTTTAAATCATAAATGTCGAATGA		
***sei***	Sei-F	CTCAAGGTGATATTGGTGTAGG	529	[33]
	Sei-R	CAGGCAGTCCATCTCCTGTA		
***sej***	Sej-F	CAGCGATAGCAA AAA TGA AAC A	426	[35]
	Sej-R	TCTAGCGGA ACA ACAGTTCTG A		
***sek***	Sek-F	CGCTCAAGGCGATATAGGAA	570	[36]
	Sek-R	GGTAACCCATCATCTCCTGTGT		
***sel***	Sel-F	CACCAGAATCAC ACCGCT TA	240	[33]
	Sel-R	CTGTTTGATGCTTGCCATTG		
***sem***	Sem-F	CTATTAATCTTTGGGTTAATGGAGAAC	300	[23]
	Sem-R	TTCAGTTTCGACAGTTTTGTTGTCAT		
***sen***	Sen-F	ATGAGATTGTTCTACATAGCTGCAAT	680	[23]
	Sen-R	AACTCTGCTCCCACTGAAC		
***seo***	Seo-F	AGTTTGTGTAAGAAGTCAAGTGTAGA	180	[23]
	Seo-R	ATCTTTAAATTCAGCAGATATTCCATCTAAC		
***sep***	Sep-F	GAATTGCAGGGAACTGCTTT	537	[36]
	Sep-R	ACCAACCGAATCACCAGAAG		
***seq***	Seq-F	GAACCTGAAAAGCTTCAAGGA	509	[36]
	Seq-R	CCAGTTCCGGTGTAAAACAAA		
***ser***	Ser-F	TTCAGTAAGTGCTAAACCAGATCC	367	[37]
	Ser-R	CTGTGGAGTGCATTGTAACGCC		
***seu***	Seu-F	ATGGCTCTAAAATTGATGGTTCTA	409	[37]
	Seu-R	GCCAGACTCATAAGGCGAACTA		
***tsst***	Tsst-F	TGCAAAAGCATCTACAAACGA	499	[36]
	Tsst-R	TGTGGATCCGTCATTCATTG		
***eta***	Eta-F	ACTGTAGGAGCTAGTGCATTTGT	190	[23]
	Eta-R	TGGATACTTTTGTCTATCTTTTTCATCAAC		
***etb***	Etb-F	CAGATAAAGAGCTTTATACACACATTAC	612	[23]
	Etb-R	AGTGAACTTATCTTTCTATTGAAAAACACTC		
***etd***	Etd-F	CGCAAATACATATGAAGAATCTGA	452	[38]
	Etd-R	TGTCACCTTGTTGCAAATCTATAG		

### Pulsed-field gel electrophoresis (PFGE)

PFGE analysis was performed for all the *S. aureus* isolates. The procedures and buffers used for the preparation of chromosomal DNA, macro-restriction of the DNA, and PFGE were modified from an earlier report [29]. Briefly, 50 µl cell suspensions were mixed with equal volumes of 1.6% low-melting-point agarose (BioProducts, Rockland, ME) and then dispersed into plug molds. After solidification at 4°C, the plugs were transferred into 2 ml positive lysis buffer containing 1 mg/ml lysostaphin and 50 µg/ml lysozyme (Sigma-Aldrich, St Louis, MO), and incubated overnight at 37°C without shaking. After incubation, the positive lysis buffer was removed and replaced with 2 ml negative lysis buffer containing 50 µg/ml proteinase K (Sigma-Aldrich). The plugs were incubated overnight again at 55°C and then washed 3x with Tris-EDTA buffer. After that, the plugs were transferred into 2 ml of fresh TE buffer and stored at 4°C until further analysis.

For restriction digestion, a plug was cut into small slices and placed in a 100 µl reaction mixture containing 20 U of *Sma*I (New England BioLabs, Beverly, MA). After a 16 hr incubation at 30°C, digested chromosomal fragments were analyzed by loading the trimmed slices of the plug into a well of a 1% SeaKem agarose gel (FMC Corp. Rockland, ME) prepared with 0.5x TBE buffer (44.5 mM Tris-borate and 1 mM EDTA, pH 8.0). All the wells containing plug slices were sealed with 0.8% SeaPlaque agarose. Electrophoresis was performed in the CHEF-DR III electrophoresis cell (Bio-Rad, Melville, NY) under the following conditions: initial pulse of 5 sec, final pulse of 45 sec, at 6 V/cm at 14°C for 24 hrs. After staining with ethidium bromide and destaining with ddH_2_O, the gel was visualized using a UV transilluminator. Lambda DNA (New England Biolabs) was used as size standards and served as a control for the running parameters of the CHEF-DR units. The patterns of DNA fingerprint were analyzed using BioNumerics 4.0 software (Applied Maths NV, Austin, TX). The band patterns among different strains were compared using Dice coefficients with a 1.5% band position tolerance. A dendrogram of PFGE results was created using the unweighted pair group method with arithmetic averages (UPGMA). The cluster cutoff was set at 80% similarity [30].

### Multilocus sequence typing (MLST)

Amplification of 7 housekeeping genes (*arcC*, *aroE*, *glpF, gmk, pta, tpi* and *yqiL*) of *S. aureus* was performed using the primers and PCR conditions specified at the MLST website (http://www.mlst.net). Amplified PCR fragments were visualized on agarose gels and purified using the Agencourt AMPure XP system (Beckman Coulter, Inc., Beverly, MA). Sequencing reactions were carried out in both forward and reverse directions using the same primers for PCR and the BigDye Terminator v3.1 cycle sequencing kit (Applied Biosystems) [31]. The products were cleaned of residual dyes using the Agencourt CleanSEQ system (Beckman Coulter, Inc.) following the manufacturer's instructions. Nucleotide sequences were determined using an ABI 3730 DNA sequencer (Applied Biosystems). Results from each primer set were assembled using the Sequencher software v4.10 (Gene Codes Corp., Ann Arbor, MI). In a few cases, new sequencing reactions were performed and analyzed to resolve ambiguities. The DNA sequences of each locus were compared to the allele sequences in the MLST database. The alleles not matched to the corresponding sequences in the database were submitted as new registrations. The combination of alleles at the seven loci was defined as an allelic profile for each isolate, and each allelic profile was assigned to a sequence type (ST), either already defined in the database or designated as a new registration by the website curator. The eBURST algorithm was used to assign MLST clonal complexes (CCs) (http://eburst.mlst.net). In addition, the nucleotide sequences of new alleles were submitted to GenBank (http://www.ncbi.nlm.nih.gov/genbank) and given accession numbers JN585753 - JN585759.

### *agr* genotyping

*agr* allele types (I – IV) were determined by multiplex PCR using the *agr*-group specific primers and amplification conditions described by Gilot *et al*. [11]. Briefly, a common forward primer of Pan (5′-ATGCACATGGTGCACATGC-3′) was used. Reverse primers included agr1 (5′-GTCACAAGTACTATAAGCTGCGAT-3′), agr2 (5′-TATTACTAATTGAAAAGTGGCCATAGC-3′), agr3 (5′-GTAATGTAATAGCTTGTATAATAATACCCAG-3′), and agr4 (5′-CGATAATGCCGTAATACCCG-3′). These primers allow the amplifications of 441-bp, 575-bp, 323-bp, and 659-bp DNA fragments from the *agr* group I, II, III, and IV strains, respectively. Four strains whose *agr* amplicons were sequenced and confirmed to be each of the *agr* groups were used as positive controls.

## References

[1] Wertheim HF, Melles DC, Vos MC, van Leeuwen W, van Belkum A (2005). The role of nasal carriage in *Staphylococcus aureus* infections.. Lancet Infect Dis.

[2] Waters AE, Contente-Cuomo T, Buchhagen J, Liu CM, Watson L (2011). Multidrug-Resistant *Staphylococcus aureus* in US Meat and Poultry.. Clin Infect Dis.

[3] Becker K, Friedrich AW, Lubritz G, Weilert M, Peters G (2003). Prevalence of genes encoding pyrogenic toxin superantigens and exfoliative toxins among strains of *Staphylococcus aureus* isolated from blood and nasal specimens.. J Clin Microbiol.

[4] Argudín MA, Mendoza MC, Rodicio MR (2010). Food Poisoning and *Staphylococcus aureus* Enterotoxins.. Toxins.

[5] Baba T, Takeuchi F, Kuroda M, Yuzawa H, Aoki K (2002). Genome and virulence determinants of high virulence community-acquired MRSA.. Lancet.

[6] Lindsay JA, Holden MT (2006). Understanding the rise of the superbug: investigation of the evolution and genomic variation of *Staphylococcus aureus*.. Funct Integr Genomic.

[7] Kuroda M, Ohta T, Uchiyama I, Baba T, Yuzawa H (2001). Whole genome sequencing of meticillin-resistant Staphylococcus aureus.. Lancet.

[8] Novick RP, Christie GE, Penades JR (2010). The phage-related chromosomal islands of Gram-positive bacteria.. Nat Rev Microbiol.

[9] Omoe K, Hu DL, Takahashi-Omoe H, Nakane A, Shinagawa K (2003). Identification and characterization of a new staphylococcal enterotoxin-related putative toxin encoded by two kinds of plasmids.. Infect Immun.

[10] Wu D, Li X, Yang Y, Zheng Y, Wang C (2011). Superantigen gene profiles and presence of exfoliative toxin genes in community-acquired meticillin-resistant *Staphylococcus aureus* isolated from Chinese children.. J Med Microbiol.

[11] Gilot P, Lina G, Cochard T, Poutrel B (2002). Analysis of the genetic variability of genes encoding the RNA III-activating components Agr and TRAP in a population of Staphylococcus aureus strains isolated from cows with mastitis.. J Clin Microbiol.

[12] Hallin M, Deplano A, Denis O, De Mendonca R, De Ryck R (2006). Validation of pulsed-field gel electrophoresis and spa typing for long term, nation-wide epidemiological surveillance studies of *Staphylococcus aureus* infections.. J Clin Microbiol.

[13] Peacock SJ, de Silva GD, Justice A, Cowland A, Moore CE (2002). Comparison of multilocus sequence typing and pulsed-field gel electrophoresis as tools for typing *Staphylococcus aureus* isolates in a microepidemiological setting.. J Clin Microbiol.

[14] Varshney AK, Mediavilla JR, Robiou N, Guh A, Wang XB (2009). Diverse Enterotoxin Gene Profiles among Clonal Complexes of *Staphylococcus aureus* Isolates from the Bronx, New York.. Appl Environ Microbiol.

[15] Holtfreter S, Grumann D, Schmudde M, Nguyen HT, Eichler P (2007). Clonal distribution of superantigen genes in clinical *Staphylococcus aureus* isolates.. J Clin Microbiol.

[16] Takano T, Higuchi W, Otsuka T, Baranovich T, Enany S (2008). Novel characteristics of community-acquired methicillin-resistant *Staphylococcus aureus* strains belonging to multilocus sequence type 59 in Taiwan.. Antimicrob Agents Chemother.

[17] Noto MJ, Archer GL (2006). A subset of *Staphylococcus aureus* strains harboring staphylococcal cassette chromosome mec (SCCmec) type IV is deficient in CcrAB-mediated SCCmec excision.. Antimicrob Agents Chemother.

[18] Chen CJ, Huang YC (2005). Community-acquired methicillin- resistant *Staphylococcus aureus* in Taiwan.. J Microbiol Immunol Infect.

[19] Jarraud S, Lyon GJ, Figueiredo AM, Gerard L, Vandenesch F (2000). Exfoliatin-producing strains define a fourth *agr* specificity group in *Staphylococcus aureus*.. J Bacteriol.

[20] Ji G, Beavis R, Novick RP (1997). Bacterial interference caused by autoinducing peptide variants.. Science.

[21] Argudin MA, Mendoza MC, Mendez FJ, Martin MC, Guerra B (2009). Clonal complexes and diversity of exotoxin gene profiles in methicillin-resistant and methicillin-susceptible *Staphylococcus aureus* isolates from patients in a Spanish hospital.. J Clin Microbiol.

[22] Peerayeh SN, Azimian A, Nejad QB, Kashi M (2009). Prevalence of *agr* Specificity Groups Among *Staphylococcus aureus* Isolates From University Hospitals in Tehran.. Lab Med.

[23] Jarraud S, Mougel C, Thioulouse J, Lina G, Meugnier H (2002). Relationships between *Staphylococcus aureus* genetic background, virulence factors, *agr* groups (Alleles), and human disease.. Infect Immun.

[24] Campbell SJ, Deshmukh HS, Nelson CL, Bae IG, Stryjewski ME (2008). Genotypic characteristics of *Staphylococcus aureus* isolates from a multinational trial of complicated skin and skin structure infections.. J Clin Microbiol.

[25] Lowy FD, Aiello AE, Bhat M, Johnson-Lawrence VD, Lee MH (2007). *Staphylococcus aureus* colonization and infection in New York State prisons.. J Infect Dis.

[26] Brosius J, Dull TJ, Sleeter DD, Noller HF (1981). Gene organisation and primary structure of a ribosomal DNA operon from *Escherichia coli*.. J Mol Biol.

[27] Fan YL, Pan F, Paoli GC, Xiao YH, Sheng HH (2008). Development of a multiplex PCR method for detection of the genes encoding 16S rRNA, coagulase, methicillin resistance and enterotoxins in *Staphylococcus aureus*.. J Rapid Methods Autom Microbiol.

[28] Tang JN, Shi XM, Shi CL, Chen HC (2006). Characterization of a duplex polymerase chain reaction assay for the detection of enterotoxigenic strains of *staphylococcus aureus*.. J Rapid Methods Autom Microbiol.

[29] Bannerman TL, Hancock GA, Tenover FC, Miller JM (1995). Pulsed-field gel electrophoresis as a replacement for bacteriophage typing of *Staphylococcus aureus*.. J Clin Microbiol.

[30] Harbottle H, White DG, McDermott PF, Walker RD, Zhao S (2006). Comparison of multilocus sequence typing, pulsed-field gel electrophoresis, and antimicrobial susceptibility typing for characterization of *Salmonella enterica* serotype Newport isolates.. J Clin Microbiol.

[31] Irwin P, Nguyen LH, Chen CY, Paoli G (2008). Binding of nontarget microorganisms from food washes to anti-*Salmonella* and anti-*E. coli* O157 immunomagnetic beads: most probable composition of background Eubacteria.. Anal Bioanal Chem.

[32] Sharma NK, Rees CE, Dodd CE (2000). Development of a single-reaction multiplex PCR toxin typing assay for *Staphylococcus aureus* strains.. Appl Environ Microbiol.

[33] Cremonesi P, Luzzana M, Brasca M, Morandi S, Lodi R (2005). Development of a multiplex PCR assay for the identification of *Staphylococcus aureus* enterotoxigenic strains isolated from milk and dairy products.. Mol Cell Probes.

[34] Johnson WM, Tyler SD, Ewan EP, Ashton FE, Pollard DR (1991). Detection of genes for enterotoxins, exfoliative toxins, and toxic shock syndrome toxin 1 in *Staphylococcus aureus* by the polymerase chain reaction.. J Clin Microbiol.

[35] Rosec JP, Gigaud O (2002). Staphylococcal enterotoxin genes of classical and new types detected by PCR in France.. Int J Food Microbiol.

[36] Srinivasan V, Sawant AA, Gillespie BE, Headrick SJ, Ceasaris L (2006). Prevalence of enterotoxin and toxic shock syndrome toxin genes in *Staphylococcus aureus* isolated from milk of cows with mastitis.. Foodborne Pathog Dis.

[37] Hwang SY, Kim SH, Jang EJ, Kwon NH, Park YK (2007). Novel multiplex PCR for the detection of the *Staphylococcus aureus* superantigen and its application to raw meat isolates in Korea.. Int J Food Microbiol.

[38] Nakaminami H, Noguchi N, Ikeda M, Hasui M, Sato M (2008). Molecular epidemiology and antimicrobial susceptibilities of 273 exfoliative toxin-encoding-gene-positive *Staphylococcus aureus* isolates from patients with impetigo in Japan.. J Med Microbiol.

